# Target renal damage: the microvascular associations of increased aortic stiffness in patients with COPD

**DOI:** 10.1186/1465-9921-14-31

**Published:** 2013-03-05

**Authors:** Michelle John, Samia Hussain, Andrew Prayle, Rebecca Simms, John R Cockcroft, Charlotte E Bolton

**Affiliations:** 1 Nottingham Respiratory Research Unit, University of Nottingham, Clinical Sciences Building, City Hospital Campus, Hucknall road, Nottingham NG5 1PB, UK; 2 Department of Child Health, University of Nottingham, QMC Campus, Nottingham NG7 2UH, UK; 3 Wales Heart Research Institute, Cardiff University, Heath Park, Cardiff CF14 4XN, UK

**Keywords:** Arterial stiffness, Biomarkers, Haemodynamics, Kidney, Renal, Microvascular

## Abstract

**Background:**

Although renal impairment has been described in COPD, there is opportunity to evaluate further to determine nature and consider optimal management. Increased aortic stiffness, as seen in COPD, leads to reduced buffering of pulsatile flow. We hypothesised that urinary albumin creatinine ratio (UACR) would reflect glomerular damage related to aortic stiffness.

**Methods:**

Patients with COPD and controls underwent spirometry, blood pressure, arterial stiffness - aortic pulse wave velocity (PWV) and provided a spot urine sample for UACR, with other renal biomarkers measured.

**Results:**

The UACR was increased in patients (n = 52): 0.80 mg/mmol compared to controls (n = 34): 0.46 mg/mmol, p < 0.05. Aortic PWV was related to log_10_ UACR in all subjects (r = 0.426, p < 0.001) and COPD patients alone. Aortic PWV was a significant variable for UACR with oxygen saturations, after accounting for potential confounders. Eight subjects (7 patients) reached a defined clinical microalbuminuria threshold, with aortic PWV greater in these patients compared to those patients without, although albuminuria is a continuum. Proximal tubular damage biomarkers, unlike the glomerular marker, were not different between patients and controls.

**Conclusions:**

There is glomerular damage in patients with COPD evidenced by increased UACR, related to increased aortic stiffness. Besides the macrovascular prognostic implications of increased aortic stiffness, the microvascular state in COPD management should be considered.

## Background

Cardiovascular (CV) risk in chronic obstructive airways disease (COPD) patients has been the interest of much recent research, since CV disease contributes significantly to the increased mortality associated with COPD [1]. Often subclinical, increased aortic stiffness, [2,3] altered haemostasis, [4] endothelial and ventricular dysfunction are likely contributing factors [3,5,6].

In addition to the macrovascular events associated with increased aortic stiffness, there is also the microvasculature association [7]. The renal and cerebral vascular beds are particularly susceptible to the increased pulsatile flow they are exposed to due to the reduced buffering capacity of the large arteries, such as the aorta, associated with increased arterial stiffness. Albuminuria reflects increased permeability of the glomerulus, usually due to microvascular damage. Indeed, albuminuria has been related to all-cause mortality as well as CV events [8,9], and suggested as an early prognostic CV marker. It has been related to arterial stiffness in subjects with diabetes and hypertension [10-12], as well as in a non-diabetic, non-hypertensive population [13], and the general population [14].

The proportion of patients with COPD having co-existent renal impairment has been highlighted [15,16]. However, identification of renal disease on serum creatinine or creatinine based estimated glomerular filtration rate (eGFR) alone is likely to underestimate the prevalence; further, the eGFR role in an older population has been questioned. Casanova et al. [17] demonstrated increased urinary albumin creatinine ratio (UACR) in patients with COPD and that microalbuminuria was frequent compared to smokers; [17] being related to hypoxaemia and systolic blood pressure. However, age was markedly different between the patients and controls – which can affect UACR.

Advances in specific renal biomarkers provide an opportunity to explore processes further. Neutrophil gelatinase-associated lipocalin (NGAL) and kidney injury molecule −1 (KIM-1) are markers of proximal tubular damage; whilst serum cystatin C is a measure of glomerular function, with the advantage that unlike serum creatinine, cystatin is produced by every nucleated cell and is only eliminated by the kidney.

We hypothesised that in addition to the prognostic information that increased aortic stiffness poses for future cardiovascular events; the increased aortic stiffness in patients with COPD relates to subclinical glomerular injury.

## Methods

### Subjects

Subjects with a smoking history, with and without COPD, over 40 years of age and of European ancestry were recruited. All subjects gave written informed consent and the study was performed in accordance with the Declaration of Helsinki and approved by Leicester, Nottingham and Rutlands Research Ethics Committee (NRES ref 10/H0406/65). Subjects were excluded if known alpha-1-antitrypsin deficiency, active or suspected malignancy or terminal disease. We deliberately did not exclude subjects with ischaemic heart disease (IHD) and diabetes in order to represent clinical practice. However, we opted *a priori* to compare controls and patients with COPD in the subgroup without evidence of IHD or diabetes. All subjects had smoked greater than 10 pack years. Controls had no evidence of airways obstruction or history of respiratory disease. COPD patients were studied at clinical stability, defined as no change in regular therapy, no requirement for antibiotics and/or oral corticosteroids in the preceding 4 weeks and no change in symptoms beyond day-day variation. Subjects were recruited from departmental databases of volunteers and by poster and local newspaper advert. Further, patients were also invited from local out-patient COPD clinics.

### Cardiovascular measurements

Patients were asked to refrain from short acting bronchodilators for four hours prior to the study. Tests were performed after a period of resting supine for at least 10 minutes. Heart rate (HR) and peripheral blood pressure (BP) were performed (Omron 705IT, UK). Pulse pressure (PP) and mean arterial pressure (MAP) were calculated. Aortic pulse wave velocity (PWV) was performed using Vicorder (Skidmore Medical, UK). Sequentially recorded ECG-gated carotid and femoral artery waveforms allowed calculation of wave transit time by the system software, using the R wave of a simultaneously recorded ECG as a reference frame. Aortic PWV was determined by dividing path length by wave transit time. Pulse wave analysis (PWA) was used to generate a corresponding central waveform. With the integral software, augmentation index (AIx) was calculated as the difference between the second and first systolic peaks as a percentage of PP. Stroke Volume was also calculated through integral software and corrected for body surface area using the Mosteller calculation.

### Anthropometry and lung function

Height and weight were measured and body mass index (BMI) calculated. Fat free mass (FFM) was calculated using bioelectrical impedance with the subject encouraged to void immediately prior to assessment (Tanita 418). A low BMI was defined as <20 kg/m^2^; a low FFMI was defined as <15 kg/m^2^ in females and <16 kg/m^2^ in males. Post-bronchodilator spirometry was performed (Microlab MK6, Micromedical, UK) to determine forced expiratory volume in one second (FEV_1_), forced vital capacity (FVC) and the ratio FEV_1_/FVC. The Global initiative for chronic Obstructive Lung Disease (GOLD) stage of airflow obstruction was used to classify patients into GOLD stages I-IV. [18] Oxygen saturations after 10 minutes rest (Konica Minolta Pulsox-300) breathing air and exhaled carbon monoxide levels were performed (Clement Clarke).

### Biochemistry

Venous blood was taken for urea, electrolytes, creatinine, total cholesterol, HDL-cholesterol and triglycerides. Spot morning urine sample was collected for urinary albumin and creatinine to determine UACR. A clinical microalbuminuria threshold of 2.5 mg/mmol males and 3.5 mg/mmol females was used; although there is growing awareness that albuminuria is a continuum. All analytes were measured on the Olympus AU2700 platform (Beckman Coulter, USA) according to manufacturer’s settings. Creatinine was measured using the traditional O’Leary Beckman Coulter assay. The eGFR was determined using Modification of Diet in Renal Disease (MDRD) formula [19]. LDL-cholesterol levels were estimated using the Friedewald equation [20]. All methods had been fully quality controlled prior to analysis in a CPA accredited laboratory.

Serum was centrifuged, aliquoted and stored at −80°C for later determination of cystatin and C-reactive protein (CRP) (R&D systems, UK). Urine aliquots were stored at −80°C for later determination of NGAL and KIM-1 (R&D systems, UK). All research ELISA’s were performed in duplicate in batches. NGAL and KIM-1 were standardised for urine concentration according to urinary creatinine.

### Other measurements

Detailed history including medication and smoking were recorded. The COPD assessment tool (CAT) and St George’s Respiratory Questionnaire (SGRQ) were performed [21,22].

### Statistics

Data was analysed using Statistical Package for the Social Sciences (SPSS, Chicago, IL) version 19.0. Positively skewed data was log_10_transformed in order to perform parametric analysis where possible – UACR, CRP, NGAL and KIM-1. Results for these variables are presented as geometric mean and SD. Analyses included chi-squared test for categorical data such as sex, independent t test for comparisons between two groups of continuous data, Pearson’s correlations to determine the nature of associations and stepwise multiple regression (see results section for variables). A p < 0.05 was considered significant.

## Results

The age, sex proportion and BMI were not different, Table 1. Resting oxygen saturations of <92% on air were present in 3 patients. There were 4 patients with a low BMI but no controls, and 13 patients and 9 controls were classed as obese (BMI >30 kg/m^2^). FFMI measurement was not possible in every subject using BIA but 8/47 and 0/30 controls had a low FFMI. No subjects had a former diagnosis of renal disease and none had macroalbuminuria. There were 5 patients and 1 control that were on ACE inhibitors; 2 patients and 0 controls on beta blockers. CRP was similar between both groups, Table 2.

**Table 1:** Subject demographics

	**Control smoker**	**COPD**	**P value**
**n**	34	52	
**Age (years)**	66(11)	68 (8)	0.3
**Gender male:female**	17:17	31:21	0.38
**Smoking status current:ex**	9:25	13:39	0.88
**Smoking pack years**	29(18)	39 (21)	0.017
**FEV**_**1 **_**(L)**	2.6(0.7)	1.5 (0.6)	<0.001
**FEV**_**1 **_**% predicted (%)**	99 (13)	59(20)	<0.001
**FVC (L)**	3.6(1.1)	3.1(1.0)	0.05
**FVC % predicted (%)**	109(16)	95 (20)	0.001
**Oxygen saturations (%)**	96(1)	95 (2)	0.001
**Carbon monoxide (ppm)**	9(10)	7(8)	0.47
**CAT score**	5(5)	17(10)	<0.001
**SGRQ**	7(7)	37(21)	<0.001
**GOLD stage of airflow obstruction 1/2/3/4 (n)**	0/0/0/0	5/33/10/4	
**BMI (kg/m**^**2**^**)**	27.0 (3.4)	26.3(4.8)	0.44
**FFMI (kg/m**^**2**^**) n = 77**	18.8 (2.3)	18.2 (2.5)	0.31
**IHD (n)**	2	9	0.12
**Diabetes (n)**	4	5	0.75

All results are presented as mean (SD) unless otherwise stated.

**Abbreviations: FEV**_**1**_ forced expiration in 1 second; **FVC** forced vital capacity; **CAT** COPD assessment Test; **SGRQ** St George’s respiratory questionnaire; **GOLD** Global initiative for chronic Obstructive Lung Disease; **BMI** body mass index; **FFMI** fat free mass index; **IHD** ischaemic heart disease.

**Table 2:** Renal and inflammatory biomarkers

	**Control smoker**	**COPD**	**P value**
**Urinary KIM-1 / Cr † ng/g**	513 (2)	575 (2)	0.423
**Urinary NGAL / Cr † ng/g**	16982 (2)	16218 (2)	0.804
**Serum Cystatin C ng/ml**	818 (295)	874 (258)	0.358
**CRP † mg/ ml**	1.1 (2.3) n = 34	1.4 (2.3) n = 49	0.110

All results are presented as mean (SD) unless otherwise stated.

† Geometric Mean.

**Abbreviations: KIM-1** Kidney injury molecule 1; **NGAL** Neutrophil gelatinase-associated lipocalin; **Cr** Creatinine; **CRP** C reactive protein.

Subgroup analysis in subjects without diabetes or IHD was performed: n = 40 patients and n = 29 controls. Again, age, gender, BMI, MAP and smoking status were similar between both groups.

### Renal biomarkers

**Figure 1:**
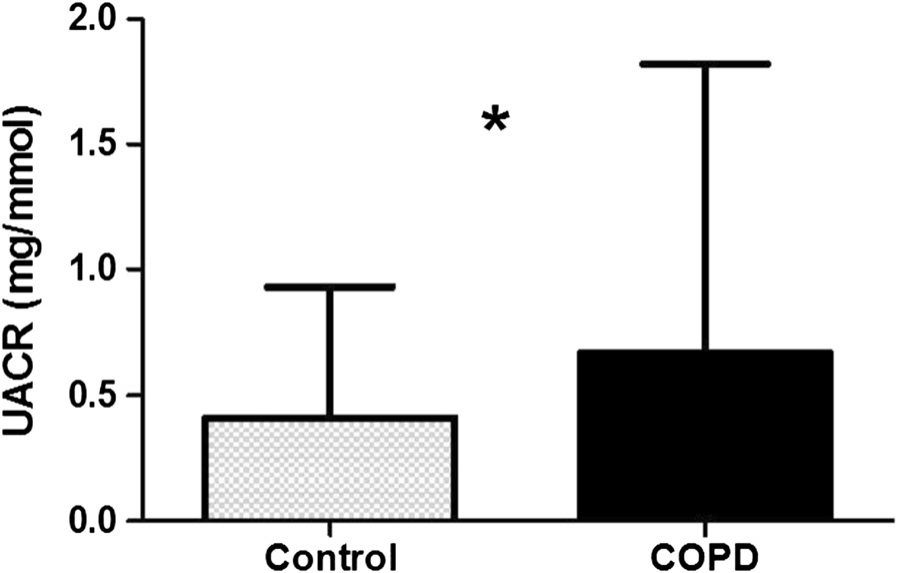
**Urinary albumin creatinine ratio in all patients with COPD and controls.** Median and IQR.

The mean eGFR was similar in patients and controls with a similar proportion: 15/52 (29%) and 10/34 (29%) having a low eGFR (<60 mls/min) based on serum creatinine. The UACR was greater in patients than controls, p < 0.05, Table 3 and Figure 1. In the subgroup without diabetes or IHD, again UACR was greater in the patients than controls: geometric mean (SD) 0.82 (3.76) and 0.45 (3.55) mg/mmol respectively, p = 0.06. Age was related to log_10_UACR, r = 0.321, p = 0.003 in the whole group and subgroup without diabetes or IHD, r = 0.357, p = 0.003. Further, in the whole population, log_10_UACR was inversely related to FEV_1_% predicted in all subjects (r = −0.231, p = 0.033) but not in COPD alone and to oxygen saturations in all subjects (r = −0.284, p = 0.008) and patients with COPD alone (r = −0.294, p = 0.03). It was not related to smoking pack years, BMI or FFMI.

**Table 3:** Haemodynamic status and biochemistry

	**Control smoker**	**COPD**	**P value**
**eGFR (ml/min)**	67 (12)	67 (14)	0.91
**UACR † (mg/mmol)**	0.46 (3.24)	0.80 (3.66)	0.046
**Aortic PWV (m/s)**	9.9 (2.3)	10.8 (1.9)	0.051
**Peripheral Systolic BP (mmHg)**	150 (22)	144 (20)	0.147
**Peripheral Diastolic BP (mmHg)**	87 (11)	83 (11)	0.102
**Peripheral Pulse pressure (mmHg)**	63 (17)	60 (17)	0.497
**Peripheral MAP (mmHg)**	108 (13)	104 ( 12)	0.078
**Heart rate (bpm)**	69 (11)	77 (17)	0.014
**AIx (%)**	21 (6)	21 (15)	0.957
**Central Pulse Pressure (mmHg)**	56 (15)	54 (14)	0.65
**Central MAP (mmHg)**	106 (14)	104 (12)	0.67
**Stroke Volume Index (ml/m**^**2**^**)**	44.9 (13.9)	44.9 (10.7)	0.99
**Total Cholesterol (mmol/L)**	5.0 (1.2)	5.1 (1.1)	0.613
**LDL-Cholesterol (mmol/L)**	2.5 (1.0)	2.7 (1.0)	0.618
**HDL-Cholesterol (mmol/L)**	1.6 (0.4)	1.8 (0.5)	0.058

All results are presented as mean (SD) unless otherwise stated.

† geometric mean.

**Abbreviations**: **eGFR** estimated glomerular filtration rate; **UACR** urinary albumin creatinine ratio; **IHD** ischaemic heart disease; **PWV** pulse wave velocity; **BP** blood pressure; **MAP** mean arterial pressure; **AIx** augmentation index.

There were 7 (13%) patients with COPD and 1 (3%) smoker control who had a UACR consistent with a clinical threshold for microalbuminuria. Of these 8, 1 was a patient with COPD who had co-existent diabetes. The other 7 had no history of diabetes or IHD.

The proximal tubule urinary markers of KIM-1/Cr and NGAL/Cr were not different between all controls and patients with COPD, Table 2. Cystatin C was not different between all patients with COPD and controls. Log_10_ cystatin C was related to eGFR (r = −0.476, p < 0.001); a greater cystatin C suggesting functional impairment.

### Haemodynamics

Aortic PWV was greater in patients than controls despite similar MAP, Table 3. In the subgroup without a past history of diabetes or IHD, the mean (SD) aortic PWV was greater in patients, 10.9 (1.7) m/s compared to controls, 9.6 (2.2) m/s, p = 0.011. In the whole population, derived stroke volume index was similar between groups.

**Figure 2:**
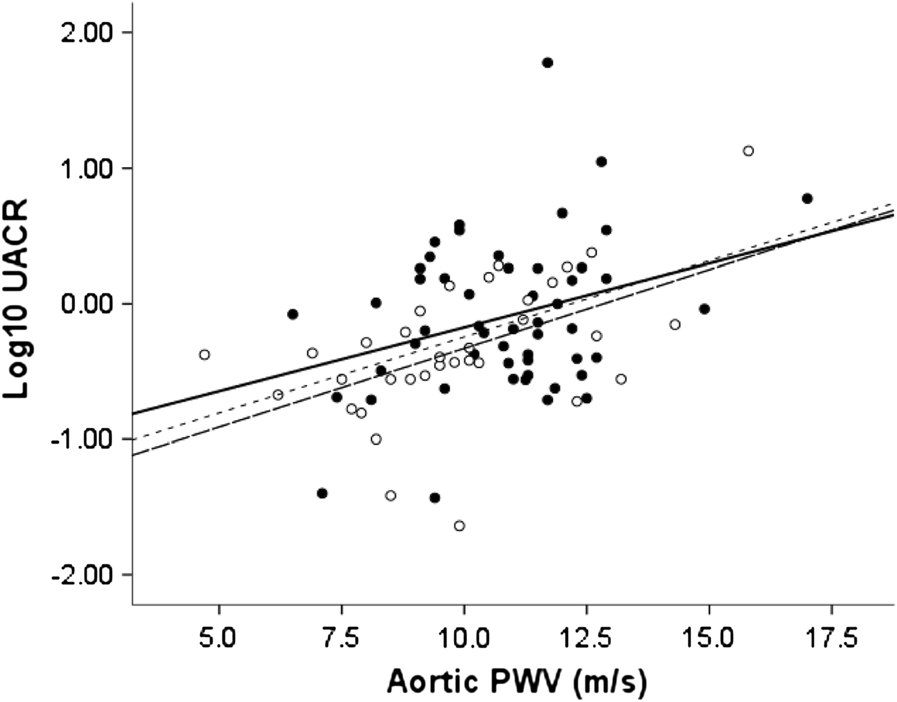
**Association of urinary albumin creatinine ratio to aortic stiffness in patients with COPD and controls.** Solid Circle – COPD, Open Circle – Control smokers, Dotted line – All subjects (r = 0.426, p < 0.001), Black line – COPD (r = 0.316, p = 0.022), Dashed line - Control smokers (r = 0.524, p = 0.001).

Log_10_ UACR was related to aortic PWV in all subjects (r = 0.426, p < 0.001) and in COPD patients alone (r = 0.316, p = 0.022), Figure 2. The association of log_10_UACR with aortic PWV, FEV_1_% predicted and oxygen saturations were also present in the subgroup without IHD or diabetes: aortic PWV r = 0.494, p < 0.001; FEV_1_% predicted r = −0.285, p = 0.018; oxygen saturations r = −0.326, p = 0.007.

Multiple stepwise regressions were performed with log_10_UACR as the dependent and age, gender, FEV%, peripheral MAP, BMI, smoke pack years, aortic PWV, oxygen saturations, IHD and diabetes as the independent variables, Table 4. In all subjects and in patients with COPD alone both aortic PWV and oxygen saturations were significant variables. The same held when peripheral MAP was substituted for peripheral PP or central MAP or central PP.

**Table 4:** **Results of stepwise multiple regression analysis, with Log**_**10**_**UACR as dependent variable and age, gender, FEV**_**1**_**%, MAP, BMI, smoke pack years, aortic PWV, oxygen saturations, IHD and diabetes as the independent variables**

**A) In all subjects**	** B**	**(95% CI)**	**Overall adjusted r**^**2**^
Constant	5.57	(0.16, 11.0)	
Aortic PWV	0.11	(0.06, 0.16)	
Oxygen saturations	−0.07	(−0.13, -0.02)	22.5%
**B) In COPD patients**	** B**	**(95% CI)**	**Overall adjusted r**^**2**^
Constant	6.39	(−0.16, 12.95)	
Aortic PWV	0.10	(0.02, 0.18)	
Oxygen Saturation	−0.08	(−0.15, -0.01)	15.9%

The significant independent variables are shown in the Table.

**Abbreviations: PWV** pulse wave velocity; **MAP** mean arterial pressure; **BMI** body mass index; **FEV**_**1**_**%,** forced expiratory volume in one second % predicted; **UACR** urinary albumin creatinine ratio.

Performed in A) all subjects (COPD and controls), B) patients with COPD alone.

Aortic PWV was greater in the 7 patients with microalbuminuria: 12.3 (2.4) m/s compared to the patients without: 10.6 (1.7) m/s, p = 0.023; and remained after adjustment for age and gender.

There was no relationship between log_10_CRP and aortic PWV. As expected, there was no relationship of either KIM-1 or NGAL with aortic PWV, or in deed with any of the other variables: FEV_1_% predicted or oxygen saturation in either all subjects or COPD alone.

## Discussion

COPD patients have increased UACR compared to controls which was related to the increased aortic stiffness. The UACR increase in the presence of similar biomarkers of proximal tubular damage would reinforce the glomerular damage leading to increased permeability in patients. Aortic stiffness was an independent variable of UACR in the setting of similar peripheral BP measures, with BP if anything marginally lower in patients with COPD.

The recent GOLD document has embedded that comorbidities are important to detect and manage in patients with COPD [18]. One of the co-morbidities highlighted as frequent and important was cardiovascular disease. Recommendations encourage healthcare professionals to manage the co-morbidity in general, as if the patient does not have COPD. However, this fundamentally relies on diagnosing the co-morbidity in the first instance and a robust platform of assessment to monitor response and progression. If renal changes are related to the altered haemodynamics, it is imperative that we fully evaluate the interplay of microvasculature to the macrovascular state in COPD. In COPD patients, associations of aortic stiffness with UACR were demonstrated despite only a small proportion above the threshold defining clinical microalbuminuria. The striking increased aortic PWV in those patients with newly identified microalbuminuria reinforces the clinical context. Albuminuria, though, is a continuum and measureable renal injury occurs below the arbitrary threshold for microalbuminuria [23,24].

A decision was made to include all patients with confirmed COPD, irrespective of co-existent comorbidities such as IHD and diabetes. This is a different approach to previous studies we have conducted but has been widely adopted by others. We opted *a priori* that this would a) represent a more typical COPD population seen in clinic and b) if there are subclinical changes in patients, then exclusion of a subset with prior diagnosed disease seemed arbitrary and may relate to diagnosis as opposed to presence.

Although a greater proportion of COPD patients who have a low eGFR compared to controls has been reported, the utility of a creatine-based eGFR as a measure of renal impairment in older populations is debateable, particularly in conditions associated with altered body composition [25]. The large proportion of subjects in both groups with impaired eGFR in this study is striking but likely due to the older nature of subjects. The advent of the biomarker era permits the ability to untangle potential anatomical sites for nephropathy. The similar concentration-corrected NGAL and KIM-1 proximal tubular biomarkers lend support to the increased urinary albumin being a primary glomerular injury. We understand this is the first to study these in COPD patients in such a comprehensive manner.

A number of studies have explored different formulae for cystatin C derived eGFR in a variety of populations. The most widely used in an unselected population is the Grubb equation which has been validated against the gold standard (invasive) method of plasma iohexol clearance [26]. Given none of the various equations have compared cystatin C to a gold standard in COPD we opted to present the cystatin C alone [27]. Cystatin C measures glomerular function and it is therefore not all-together unexpected that a marked difference in glomerular damage markers was seen despite similar cystatin C.

The association of glomerular damage to microvas-culopathy has been demonstrated in subjects with other conditions such as hypertension and diabetes, both conditions associated with increased aortic stiffness [28,29]. In order to interrogate whether aortic stiffness leads to microvascular damage via altered renal haemodynamics, renal doppler sonography permits calculation of the renal artery vascular resistance. Such studies have demonstrated associations of a resistive index to proteinuria in patients with chronic kidney disease with and without diabetes mellitus; [30] to albuminuria and also a measure of aortic stiffness (brachial-ankle) in 150 patients with type 2 diabetes mellitus; [31] and in patients with hypertension, a modest increase in renal resistive index was associated with a greater adjusted relative risk of albuminuria [32]. Fesler and colleagues highlighted the importance of PP (as a marker of arterial stiffness) in longitudinal change in renal decline whilst treating hypertension [33]. Conversely, there is the opposing argument in a biological system - Wang reported the utility of low grade albuminuria to determine progression to hypertension in a non-hypertensive and non-diabetic population over nearly 3 years [34]. This study confirms and highlights the issue in COPD which has not been previously demonstrated.

Increased permeability due to impaired filtration of the barrier may be due to direct podocyte injury but other potential reasons for the association may be mediated via systemic inflammation, hypoxaemia, endothelial dysfunction or increased sympathetic activation, all known to be affected in COPD [35-37]. Resting oxygen saturations were a significant variable of albuminuria in our study. This has been reported previously in patients with COPD and in other conditions and states such as with altitude in healthy volunteers [17,38]. Sympathetic activation is known to increase arterial stiffness and raises an interesting consideration that therapeutic modulation, such as with ACE inhibitors, that are CV protective, decrease sympathetic nervous system (SNS) activation and confer renal benefit might have a role in COPD. In a parallel manner, beta blockers inhibit SNS activity and have been shown to improve mortality in COPD although their effect on renal function is as yet unknown [39]. Although there was no association with circulating CRP in this study, systemic inflammation cannot be excluded as a mediator. Similarly, endothelial dysfunction and albuminuria remains a strong contender [40]. The most likely cause is the inter-play of a combination of several factors above or even foetal programming but beyond the scope of this study [41].

The relationship between arterial stiffness and atheroma is still not well understood and at present conventional risk factors, except BP and age do not account for much of the increased arterial stiffness. However, increased stiffness has many non atherosclerotic deleterious associations including increased pulsatility [42]. Identification of microvascular abnormalities such as increased UACR is important and provides an opportune and influential period to intervene prior to development of macrovascular disease [43].

### Points for consideration

When considering UACR, it is important to reflect on the spot test which is now accepted over twenty-four hour urine collection and has been validated [25]. UACR was measured contemporaneously from fresh urine [44]. The timing of urinary spot samples is often debated despite low diurnal variation–here morning samples where possible, avoiding first void were collected.

Pack years were not matched however all had a pack year history of greater than 10. All were of European ancestry as this could have affected haemodynamic and glomerular results. Unlike previous studies in patients with and without COPD, we did not demonstrate a difference in AIx as a secondary variable. The Stroke Volume index is derived and the method requires further evaluation.

## Conclusion

In conclusion, there is evidence of increased urinary albumin in patients with COPD which is independently related to aortic stiffness. As in other conditions with altered haemodynamics, identification of glomerular damage is important. The study highlights consideration regarding intervention to optimise the CV state in COPD management and the potential role of measurement of urinary albumin in routine assessment of COPD.

## Abbreviations

AIx: Augmentation index; BMI: Body mass index; BP: Blood pressure; COPD: Chronic Obstructive Pulmonary Disease; CRP: C reactive protein; CV: Cardiovascular; ECG: Electrocardiogram; eGFR: Estimated glomerular filtration rate; FEV1: Forced expired volume in 1 second; FFM: Fat free mass; FFMI: Fat free mass index; FVC: Forced vital capacity; GOLD: Global initiative for chronic Obstructive Lung Disease; HR: Heart rate; IHD: Ischaemic heart disease; KIM-1: Kidney injury molecule; MAP: Mean arterial pressure; NGAL: Neutrophil gelatinase-associated lipocalin; PP: Pulse pressure; PWA: Pulse wave analysis; PWV: Pulse wave velocity; UACR: Urinary albumin creatinine ratio.

## Competing interests

The authors declare that they have no competing interest.

## Authors’ contributions

CB is guarantor of manuscript content, and was responsible for study concept and design and manuscript draft. MJ contributed to study design, data acquisition, analysis and drafting of manuscript. SH contributed to clinical data acquisition and revised the manuscript. AP contributed to data interpretation and intellectual content of the manuscript. RS contributed to laboratory based data acquisition and manuscript review. JC was involved in study concept, design and reviewed the manuscripts intellectual content. Approval of the final manuscript to be published has been given by all authors.
